# Fine-tuning of the size of supramolecular nanotoroids suppresses the subsequent catenation of nano-[2]catenane†

**DOI:** 10.1039/d2sc07063d

**Published:** 2023-03-02

**Authors:** Hiroki Itabashi, Sougata Datta, Ryohei Tsukuda, Martin J. Hollamby, Shiki Yagai

**Affiliations:** a Division of Advanced Science and Engineering, Graduate School of Science and Engineering, Chiba University 1-33 Yayoi-cho, Inage-ku Chiba 263-8522 Japan; b Institute for Advanced Academic Research (IAAR), Chiba University 1-33 Yayoi-cho, Inage-ku Chiba 263-8522 Japan yagai@faculty.chiba-u.jp; c Department of Chemistry, School of Chemical and Physical Sciences, Keele University Keele, Staffordsgire ST55BG UK; d Department of Applied Chemistry and Biotechnology, Graduate School of Engineering, Chiba University Chiba 263-8522 Japan

## Abstract

A judicious combination of ring-closing supramolecular polymerization and secondary nucleation can hierarchically organize a diphenylnaphthalene barbiturate monomer bearing a 3,4,5-tri(dodecyloxy)benzyloxy unit into self-assembled nano-polycatenanes composed of nanotoroids. In our previous study, nano-polycatenanes of variable length have been formed uncontrollably from the monomer that provides nanotoroids with sufficiently wide inner void space wherein secondary nucleation is driven by non-specific solvophobic interaction. In this study, we found that the elongation of the alkyl chain length of the barbiturate monomer decreases the inner void space of nanotoroids while increasing the frequency of secondary nucleation. These two effects resulted in an increase in the yield of nano-[2]catenane. This unique property observed in our self-assembled nanocatenanes might be extended to a controlled synthesis of covalent polycatenanes using non-specific interactions.

## Abstract 2

The reduction in the inner diameter of the nanotoroids of a π-conjugated barbiturate monomer results in nano-[2]catenanes in a high yield due to enhanced secondary nucleation and subsequent steric suppression of further catenation.

## Graphical abstract

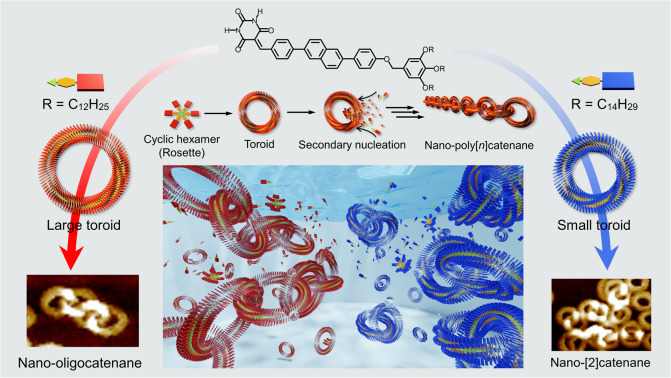

## Introduction

Nanostructures consisting of assemblies of a finite number of molecules are important for the development of nano-to-mesoscale (3–300 nm) materials with a controllable shape and size with specific properties.^1–5^ A useful strategy for obtaining such discrete nano-to-mesoscale structures is to induce curvature in one-dimensional molecular assemblies.^6,7^ Linear one-dimensional assemblies are polydisperse,^8,9^ but the introduction of a mechanism that induces an intrinsic curvature may enable efficient ring-closure. As such, nanoscale toroidal assemblies of uniform diameter can be obtained in good yield.^10–14^ These discrete toroidal assemblies (nanotoroids) can be hierarchically organized one-dimensionally by stacking into tubular assemblies^14–19^ or two-dimensionally on a substrate by densely packing into porous networks.^3,20,21^ In contrast, we have recently reported an unprecedented topological organization of toroidal assemblies by mechanically interlocking into self-assembled nanocatenanes.^22^ This sophisticated topological supramolecular organization was achieved by surface-catalyzed secondary nucleation of toroidal assemblies.^2,23–25^ The development of self-assembled nanocatenanes composed of toroidal assemblies is expected to pave the way to self-assembled materials with well-defined topologies beyond the nanoscale, *i.e.*, mesoscale.^26,27^ Toward this goal, we need to establish a method to create nano-[*n*]catenanes with a defined catenation number “*n*”.

**Scheme 1:**
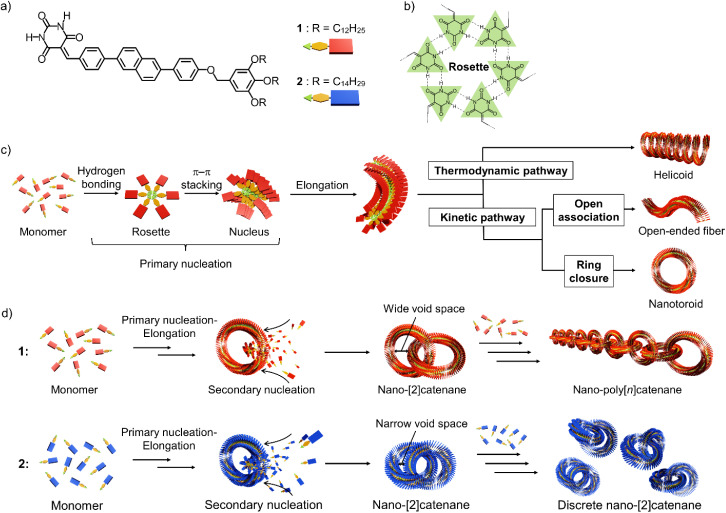
(a and b) Molecular structures of diphenylnaphthalene barbiturate monomers 1 and 2, and illustration of a hydrogen-bonded cyclic hexamer (rosette). (c) Schematic representation of the primary nucleation of barbiturate monomers leading to various topological supramolecular polymers with intrinsic curvature through thermodynamically and kinetically controlled supramolecular polymerization. (d) Schematic representation of secondary nucleation on the inner surface of 1 and 2 nanotoroids, leading to different nano-catenane species.

As the initial step in the above research direction, we herein report the suppression of nano-catenation by fine-tuning the size of nanotoroids. The nano-polycatenanes we reported previously^16^ are formed from barbituric acid molecule 1 that features a diphenylnaphthalene π-conjugated moiety and terminal dodecyl chains (Scheme 1a). This molecule forms a hydrogen-bonded hexamer called “rosettes”,^28^ and can hierarchically organize into various structures that depend on the assembly conditions (Scheme 1b and c).^6,7^ For example, slow cooling of a hot monomeric solution of 1 forms helicoidal structures *via* thermodynamically controlled supramolecular polymerization.^29,30^ Conversely, fast cooling yields toroids as kinetically trapped species.^31^ The helicoid and toroid have almost the same curvature. So these results suggested spontaneous generation of curvature in the supramolecular polymerization of 1 because rosettes continuously stack with rotational and translational displacements (Scheme 1c).^10^ Interestingly, by employing a solvent-mixing protocol that allows a more inhomogeneous kinetic aggregation, 1 furnished self-assembled catenanes.^22^ Based on the mechanistic analogy with a template-directed synthesis of molecular catenanes,^32–39^ the catenation of our nanotoroids could be attributed to secondary nucleation, *i.e.*, surface-catalyzed heterogeneous nucleation of coexisting molecules on the surface of toroids.^23^ Since the secondary nucleation occurs uniformly on the surface of the nanotoroids in an uncontrolled fashion, the current situation is that mixtures of elongated linear and branched polycatenanes are obtained with a wide distribution of *n*.

In this study, we show that a reduction in the inner diameter of the nanotoroids results in nano-[2]catenanes (*n* = 2) in a high yield due to enhanced secondary nucleation and subsequent steric suppression of further catenation (Scheme 1d).

Compound 2, analogous to 1 with dodecyl chains but with longer tetradecyl chains, forms nanotoroids with a narrow inner void space due to a slight increase in the rosettes’ stacking slip. In the nano-[2]catenanes, the additional alkyl bulk of 2*vs.*1 further narrows their inner void space, suppressing the subsequent catenation. This proposed mechanism is similar to the concept of self-interruption for the inhibition of the polymerization reaction by steric hindrance.^40^

## Results and discussion

### Method to obtain the nanocatenanes

**Fig. 1:**
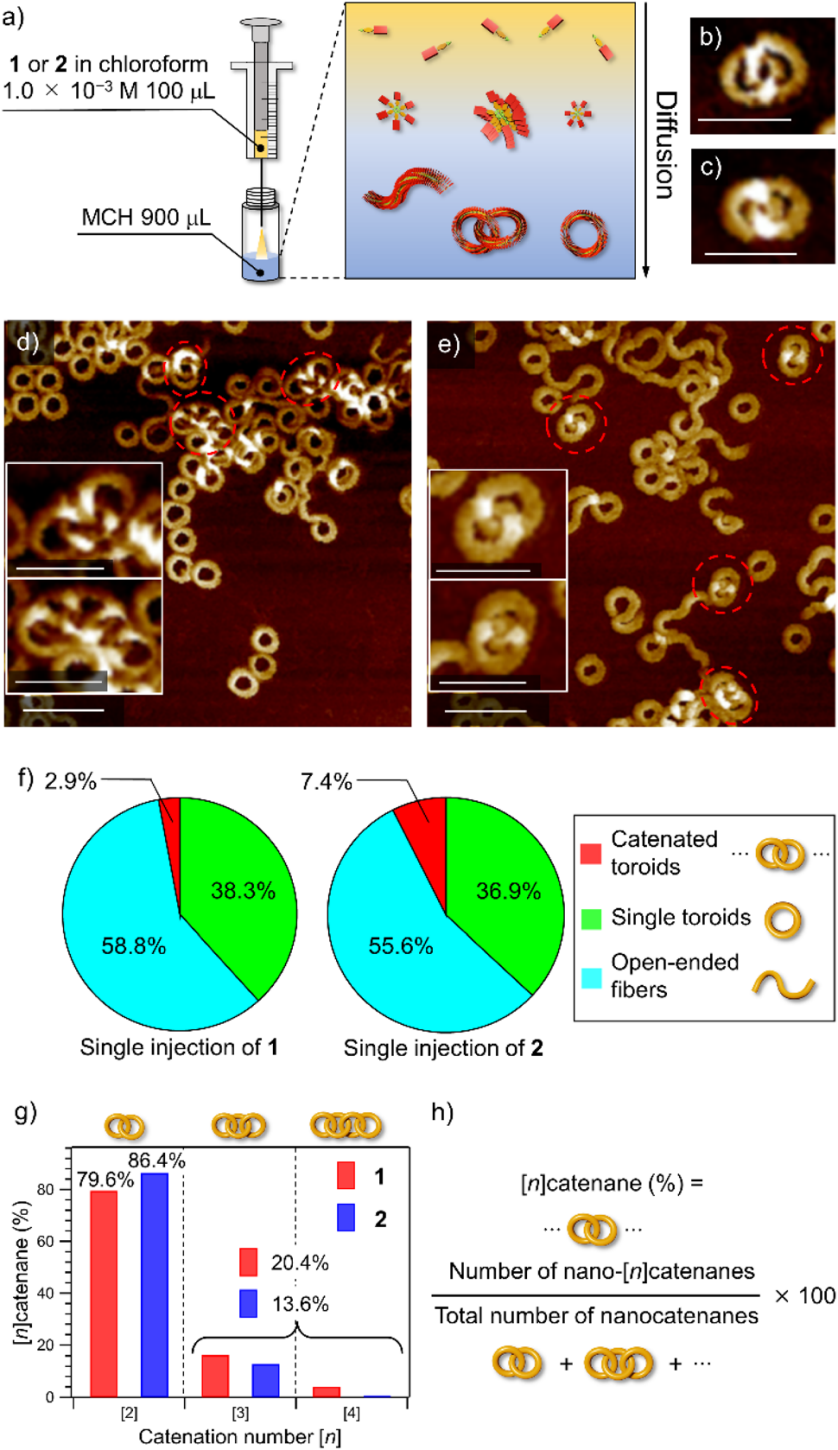
(a) Depiction of the solvent-mixing protocol. (b and c) AFM images of nano-[2]catenanes of 1 (b) and 2 (c). Scale bars, 50 nm. (d and e) AFM images of supramolecular polymers obtained by injecting a 100 μL chloroform solution (*c* = 1.0 × 10^−3^ M) of 1 (d) and 2 (e) into 900 μL of MCH in one portion. Scale bars, 50 nm in insets and 100 nm in whole images, respectively. Catenated toroids are shown by red dotted circles. (f) Pie charts showing the yields of nano-[*n*]catenanes (red), single nanotoroids (green), and open-ended fibers (light blue) obtained by injecting a 100 μL chloroform solution (*c* = 1.0 × 10^−3^ M) of 1 (left) and 2 (right) into 900 μL of MCH in one portion. The numbers of sampled nanotoroids are 1604 for 1 and 1685 for 2, respectively. (g) Bar charts showing the percentages of nano-[2], [3] and [4]catenanes of 1 (red) and 2 (blue) obtained by single-injection. (h) Formula for calculation of the percentage of nano-[*n*]catenanes.

Catenane formations through self-assembly of 1 and 2 (for the synthesis, see the ESI†) were initially investigated by vigorously injecting a concentrated chloroform (good solvent) solution of monomers (*c* = 1.0 × 10^−3^ M, 100 μL) into methylcyclohexane (poor solvent, MCH, 900 μL) to allow supramolecular polymerization under kinetic control (Fig. 1a). The resulting assemblies (total *c* = 1.0 × 10^−4^ M) were spin-coated onto highly oriented pyrolytic graphite (HOPG) and examined by AFM (Fig. 1b–e and S1, ESI†). The analysis of the acquired AFM images revealed that 2 formed nano-[*n*]catenanes in 7.4% yield based on monomers, which was higher than that of 1 (2.9%, Fig. 1f and S2, ESI†). However, the analysis of the distribution of the catenation number [*n*] revealed that 2 furnished a larger number of nano-[2]catenanes (1: 79.6% and 2: 86.4%) and a smaller number of nano-[3] and [4]catenanes compared to 1 (Fig. 1g).

### Secondary nucleation

**Fig. 2:**
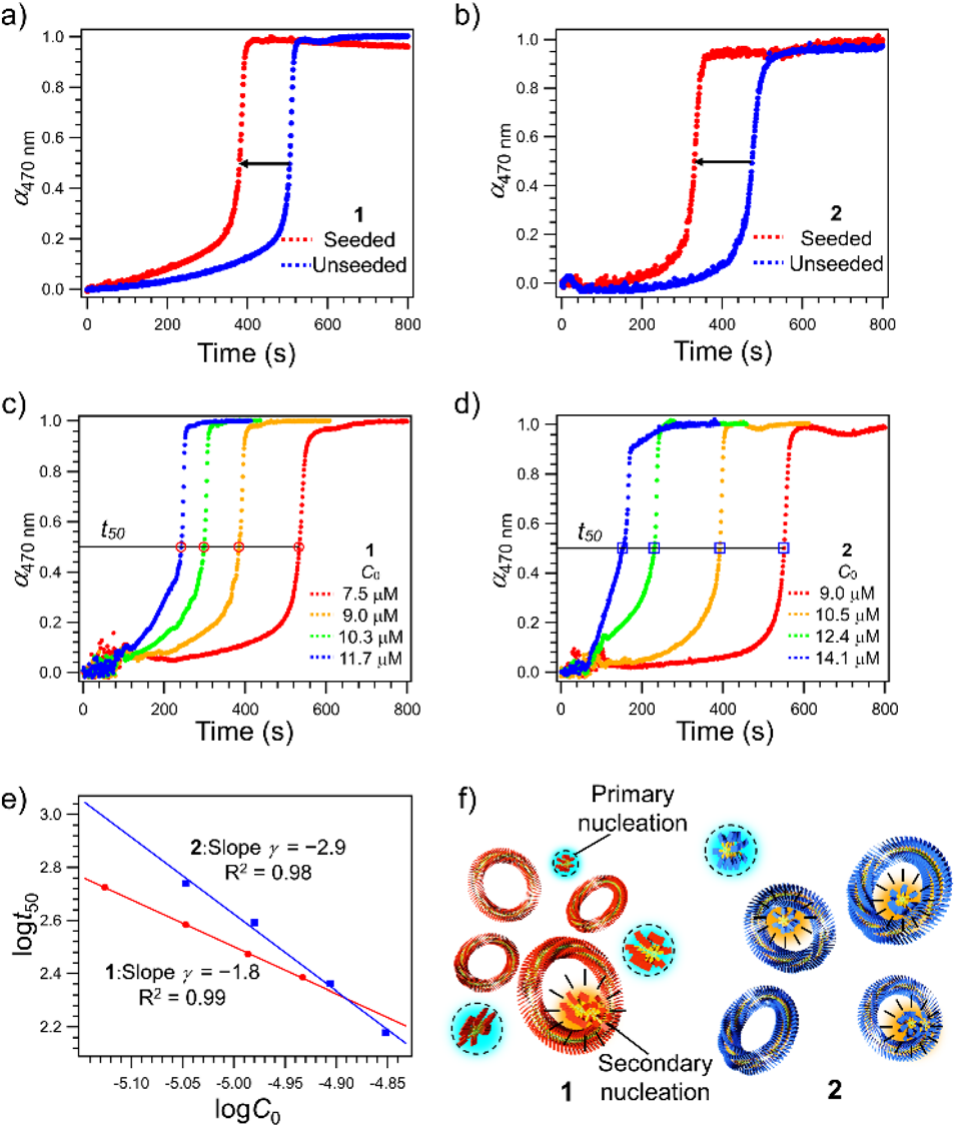
(a and b) Comparison of time-dependent changes in the molar fractions of aggregated 1 (a) and 2 (b) (*α*_470 nm_, monitored at wavelength *λ* = 470 nm) in a monomeric MCH solution (*C* = 7.5 × 10^−6^ and 7.6 × 10^−6^ M, for 1 and 2, respectively) at 333 K in the presence (red dots) and absence (blue dots) of nanotoroid seeds ([seed] = 2.4 × 10^−6^ M for both 1 and 2) with stirring at 500 rpm. (c and d) Time-dependent changes in the molar fractions of aggregated 1 (c) and 2 (d) at various *C*_0 in_ MCH at 333 K with stirring at 500 rpm in the absence of nanotoroid seeds. (e) Log–log plot of the time required for 50% decrease of *C*_0_ of 1 (red circle) and 2 (blue square) as a function of *C*_0_. Each experimental curve in (c and d) shows a representative result of at least three measurements with excellent reproducibility; the standard deviation of *γ* is 0.1 for both 1 and 2. (f) Schematic representation of secondary nucleation on the inner surface of nanotoroids of 1 (red) and 2 (blue). Primary and secondary nucleation sites are highlighted in light blue and orange colors, respectively.

Since the higher catenation yield in 2 than in 1 suggests that secondary nucleation is more likely to occur in the supramolecular polymerization of the former, we investigated the kinetics of the nucleation process of monomers in the absence and presence of purified nanotoroids as seeds.^41–43^ A detailed protocol for the purification of nanotoroids is described in the ESI† and the data are shown in Fig. S3.† Since the toroidal seeds do not have supramolecular polymer termini, this experiment can prove whether the toroid surface triggers secondary nucleation. Because it was difficult to analyze the nucleation kinetics by injection, we monitored the growth of supramolecular polymers at 333 K, which is a little lower than the nucleation temperature of 2. The growth curve of unseeded monomer solutions, obtained by monitoring the absorption band at 470 nm that is associated with the π–π stacking of the diphenylnaphthalene core, showed a sufficiently long lag time for nucleation at this temperature (Fig. S4, ESI†). On the other hand, when we added the purified toroid solution to the monomer solution kept at 353 K and then cooled the reaction mixture to 333 K, a significant shortening of the lag time was observed for both 1 and 2 (Fig. 2a and b). The degree of lag time shortening was larger in 2 (Δ*t*_50_ ≈ 140 s) than in 1 (Δ*t*_50_ ≈ 120 s), which suggests that 2 has a higher tendency towards secondary nucleation.

To analyze the contribution of secondary nucleation more quantitatively, we analyzed the aggregation kinetics of 1 and 2 in detail.^22,44^ Knowles and co-workers reported well-established models of amyloid-forming peptides and the protocol of systematic kinetic analyses.^45–47^ For the aggregation process of the amyloid-forming peptides, the half-time of the elongation kinetics (*t*_50_) is related to the initial monomer concentration (*C*_0_) by the power decay law

1 *t*_50_ ≈ *C*_0_^*γ*^

where *γ* is the scaling exponent and related to the reaction order (*n*_2_) according to the following eqn (2) if secondary pathways are responsible for the generation of new aggregates.2
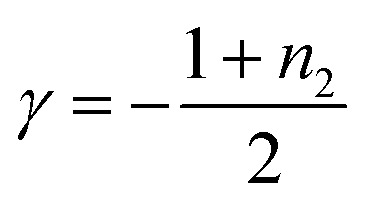


To analyze the aggregation kinetics of 1 and 2, we kept their MCH solutions of different concentrations at 333 K with stirring at 500 rpm and monitored the growth of the absorption at 470 nm that increases upon aggregation. As shown in Fig. 2c and d, sigmoidal growth kinetics with an obvious lag-phase were observed in all the measurements. We plotted log *t*_50_ as a function of log *C*_0_ to estimate *γ* values (Fig. 2e). As shown in Fig. 2e, the double-logarithmic plot resulted in a linear relationship for both 1 and 2, from which *γ* values were estimated to be −1.8 ± 0.1 for 1 and −2.9 ± 0.1 for 2, respectively. These values are consistent with the monomer-dependent secondary nucleation in the self-assembly process. From the *γ* values, *n*_2_ values are calculated using eqn (2) to be 2.6 for 1 and 4.8 for 2, respectively. This result indicates that the dependence of nucleation on monomer concentration is more pronounced for 2 than for 1, which could be attributed to the stronger intermolecular interaction associated with the longer alkyl chains (Fig. 2f). The stronger interaction between the longer alkyl chains was also manifested by precipitation of 2 in another injection experiment using a more nonpolar solvent such as *n*-octane, which was not observed for 1. Hence, the longer alkyl chains of 2 could enhance the intermolecular interaction on the surface of toroids, which is favorable for surface-catalyzed secondary nucleation. On the other hand, the critical temperature (*T*_e_) of 2 (352 K) is lower than that of 1 (356 K), suggesting a higher energy barrier for primary nucleation of 2 than that of 1 because the bulkier exterior alkyl chains of 2 inhibit the stacking of rosettes (Fig. S5, ESI†).

### Toroid size

**Fig. 3:**
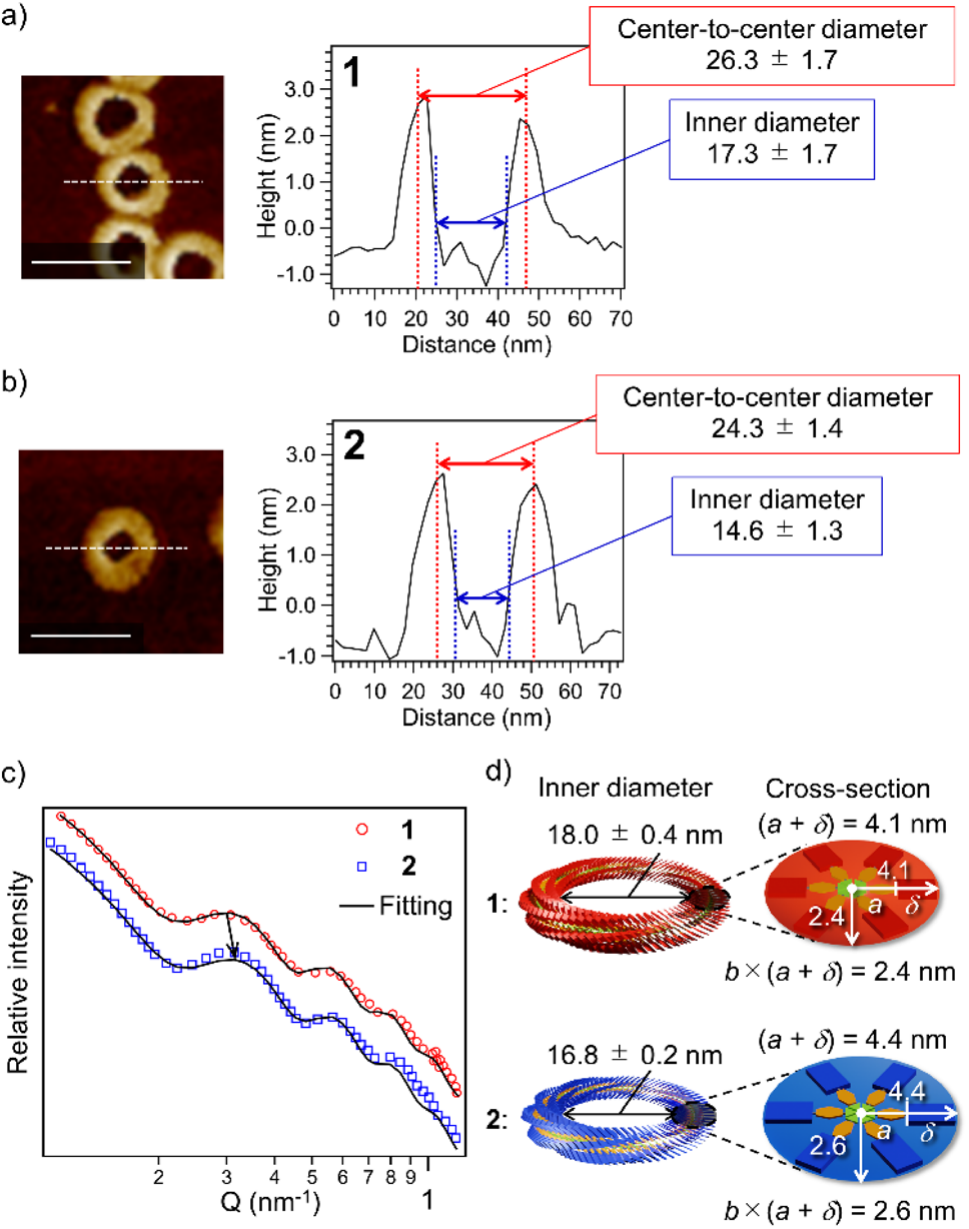
(a and b) AFM cross-sectional analysis for nanotoroids of 1 (a) and 2 (b) along the white lines. Scale bars, 50 nm. (c) SAXS profiles of purified nanotoroids of 1 (red circles) and 2 (blue squares) in MCH (*c* = 1.0 × 10^−4^ M). Black solid lines represent fits of the data using a toroid form factor and a flat background. (d) Schematic representation of size differences of rosettes and nanotoroids of 1 (red) and 2 (blue) based on the SAXS data.

The above result posed a question of why the higher catenation tendency of 2 than 1 (Fig. 1f) does not link to the increase in the proportion of nano-[*n* > 2]catenane species (Fig. 1g). The result implies that nano-[2]catenane of 2 suppresses the subsequent secondary nucleation inside the constituent nanotoroids. The larger rosette of 2 should give a narrower void space in nanotoroids (Fig. S6, ESI†). In fact, AFM cross-sectional analysis for nanotoroids of 1 and 2 (Fig. 3a and b) revealed that the average inner diameter of toroids of 2 (14.6 ± 1.3 nm) is significantly smaller than that of 1 (17.3 ± 1.7 nm). In addition, we found that the center-to-center diameter of nanotoroids of 2, which is not directly affected by the alkyl chain length extension, was also smaller than that of 1 (Table S1, ESI†). This finding suggests that alkyl chain extension can make the curvature tighter.

To further investigate the above finding, we evaluated nanotoroid sizes in solution using the small-angle X-ray scattering technique (SAXS).^48^ The SAXS profiles of purified toroids of 1 and 2 displayed similar nonperiodic oscillatory features at *Q* = 0.1–1.0 nm^−1^ (Fig. 3c). The smallest-*Q* scattering peak around 0.3 nm^−1^, which roughly corresponds to the diameter of nanotoroids, appeared at a higher *Q* value for 2 than 1, as indicated by an arrow in Fig. 3c. The nanotoroid dimensions obtained by fitting analysis using SASFit^49^ are as follows: toroid radius, *R* = 13.1 ± 0.2 nm (1) and 12.8 ± 0.1 nm (2); cross-sectional radius excluding the alkyl shell, *a* = 2.5 nm (1 and 2); aspect ratio, *b* = 0.58 (1) and 0.60 (2); alkyl shell width, *δ* = 1.6 (1) and 1.9 nm (2), respectively. From these dimensions, an internal circular void with a diameter of 18.0 ± 0.4 and 16.8 ± 0.2 nm and a fiber width of 8.2 and 8.8 nm can be estimated for 1 and 2, respectively (Fig. 3d). The smaller inner diameter (void size) of nanotoroids of 2 than that of 1 is thus not only because of its larger diameter of the supramolecular fiber but also because of the tighter curvature formed by 2 than by 1. This can be attributed to a larger rotational and translation slip of the rosettes of 2 compared to those of 1 upon stacking, as longer C_14_H_29_ chains are sterically more demanding than C_12_H_25_ chains (Scheme 1c). Accordingly, once the nano-[2]catenane is formed by 2, it prevents the subsequent catenation due to a narrow void space.

### Portion-wise injection

**Fig. 4:**
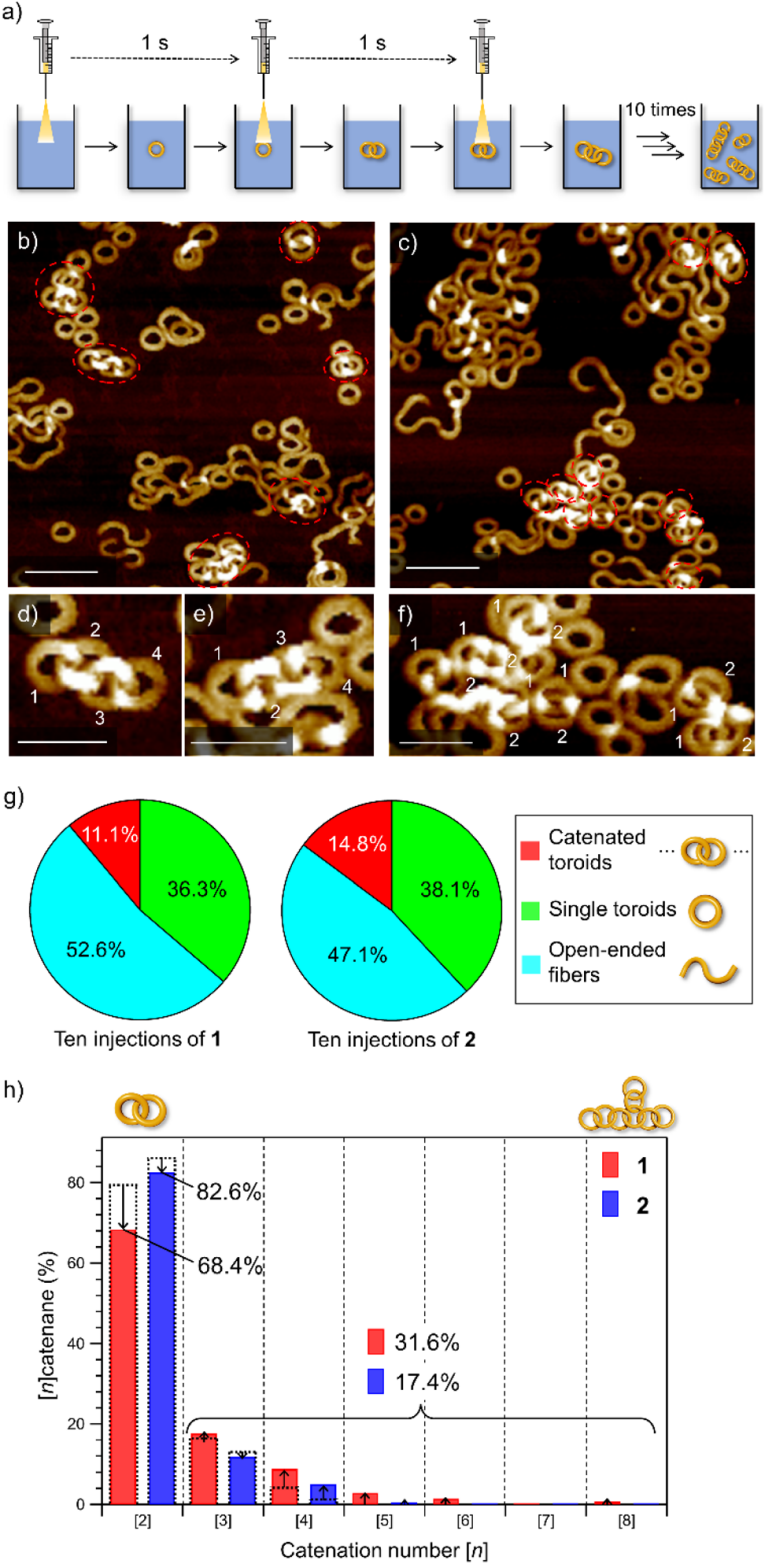
(a) Depiction of the portion-wise solvent-mixing protocol. (b and c) AFM images of supramolecular polymers obtained by injecting a 100 μL chloroform solution (*c* = 1.0 × 10^−3^ M) of 1 (b) or 2 (c) into 900 μL of MCH in ten portions (one injection per second). Scale bars, 100 nm. Catenated toroids are shown in red dotted circles. (d–f) AFM images of the nano-[4]catenane of 1 (d and e) and nano-[2]catenane of 2 (f). Scale bars, 50 nm. (g) Pie charts showing the yields of catenanes (red), single toroids (green), and open-ended fibers (light blue) in MCH from a portion-wise solvent-mixing experiment. The numbers of sampled toroids are 1478 and 1419 for 1 and 2, respectively. (h) Bar charts showing the percentages of oligomeric nano-[*n*]catenanes (*n* = 2–8) of 1 (red) and 2 (blue) obtained by portion-wise injections. Overlapped black dotted bars indicate the percentages of nano-[*n*]catenane obtained by single-injection shown in Fig. 1g.

To emphasize the low probability of further catenation in the nano-[2]catenane of 2, we conducted a portion-wise injection method. In our previous study using 1, the catenation number *n* significantly increased using this modified injection method that can elongate polycatenane chains in a living manner.^22^ In this method, a monomer chloroform solution (*c* = 1.0 × 10^−3^ M, total 100 μL) was injected into 900 μL of MCH in a portion-wise fashion (10 injections of 10 μL solution in 10 seconds, Fig. 4a).

When this method was applied to 1 and 2 in the present study, increases in the overall yield of catenated nanotoroids were observed both for 1 (2.9% → 11.1%) and 2 (7.4% → 14.8%) as revealed by AFM image analysis (Fig. 4b, c, g and S7, ESI†). The catenation number *n* was even more strongly affected by the injection method (Fig. 4d–f). By the single-injection, there was a 6.8% difference in the proportion of nano-[2]catenane between 1 and 2 (Fig. 1g). However, the difference became larger (∼15%) by applying the portion-wise injection (Fig. 4h). For 1, the decrease of nano-[2]catenane by the portion-wise injection was compensated for by an increase in the number of oligomeric nano-[*n* ≥ 3]catenanes (*n* = 3–8) from 20.4% (Fig. 1g) to 31.6% (Fig. 4h). In contrast, for 2 the increase of oligomeric nano-[*n* ≥ 3]catenanes by the portion-wise injection was only from 13.6% (Fig. 1g) to 17.4% (Fig. 4h and S7, ESI†), and nano-[*n* ≥ 6]catenanes could not be found at all.

### Inner void space

**Fig. 5:**
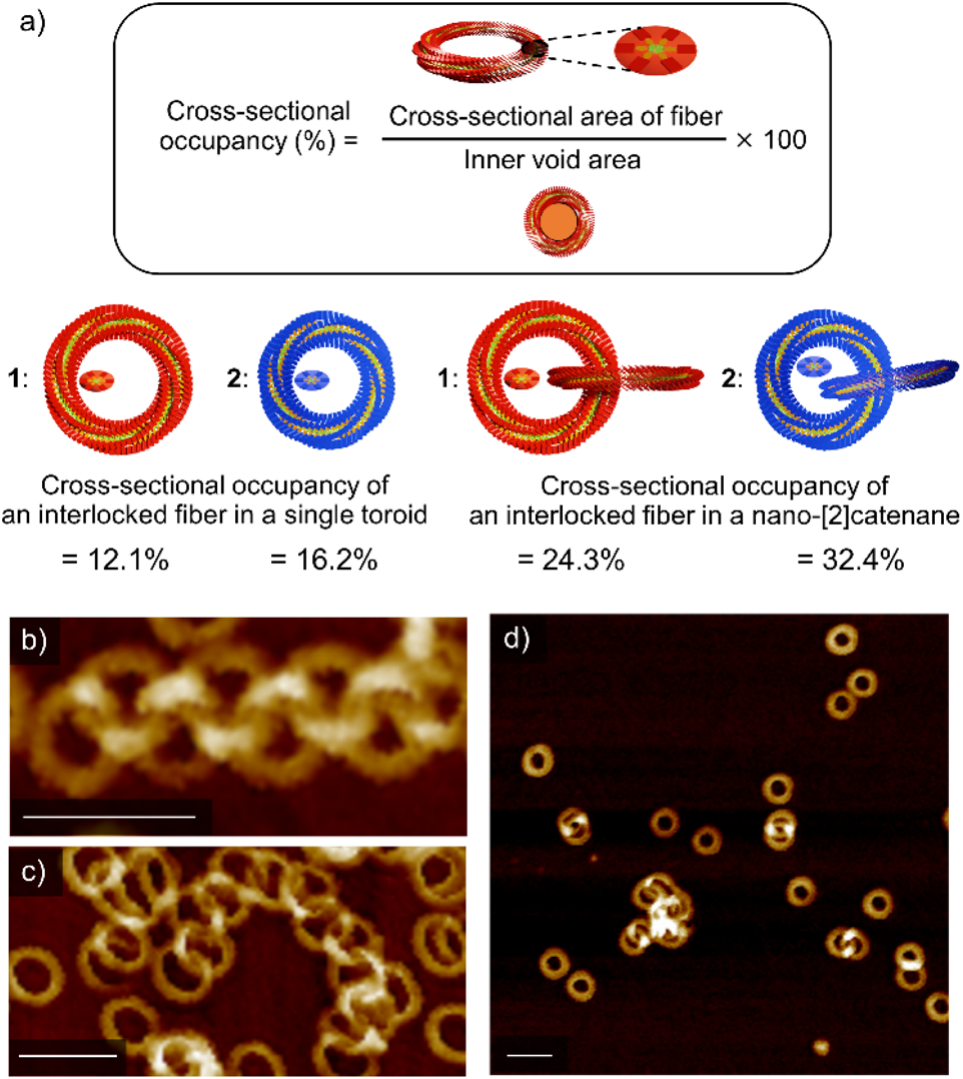
(a) Formula and representations of occupancy of an interlocked fiber of 1 (red) and 2 (blue) in the corresponding single toroid and nano-[2]catenane according to SAXS data. (b–d) AFM images of soluble nanostructures obtained by injecting a 100 μL chloroform solution (*c* = 1.0 × 10^−3^ M) of 1 (b and c) or 2 (d) into 900 μL of *n*-octane in ten portions (one injection per second). Scale bars, 50 nm.

All the above results corroborate that nano-[2]catenanes of 2 are less likely to elongate upon further feeding of monomers. The elongation of nano-[2]catenane of 1 was favored owing to enhanced intermolecular interactions in the specific nanospace provided by two interlocked toroids that facilitates secondary nucleation more effectively than the single toroids (Fig. S8, ESI†). Although the longer alkyl chains of 2 facilitate secondary nucleation as shown by the scaling experiments, the inner void space of its interlocked nanotoroids is too narrow to pass additional supramolecular fibers. Based on the SAXS data, the cross-sectional occupancies of the interlocked fiber in the inner void area of single toroids of 1 and 2 are 12.1% and 16.2%, respectively (Fig. 5a). This difference in occupancy becomes more significant in the case of nano-[2]catenanes (1: 24.3% and 2: 32.4%). The cross-sectional occupancies based on AFM data are also qualitatively consistent with those estimated from the SAXS data (Fig. S9, ESI†). Thus, the steric factor associated with the lack of sufficient void space for generating additional supramolecular fibers through secondary nucleation reduces the tendency of nano-[2]catenanes of 2 to elongate further. This effect became more pronounced when we used a more nonpolar solvent such as *n*-octane for the portion-wise injection protocols. For 1, further elongated nano-polycatenanes were observed (Fig. 5b and c). In contrast, for 2, most nanoaggregates precipitated due to enhanced solvophobic interaction associated with the formation of open-ended fibers, but only nano-[2]catenanes were detected in the solution phase (Fig. 5d).

## Conclusions

In conclusion, we demonstrated a unique methodology to control the catenation tendency for self-assembled toroidal nanoaggregates by a simple alteration of monomer structures, *i.e.*, the extension of alkyl chains that cover the nanoaggregates. Since spontaneous catenation is promoted by secondary nucleation of new nanoaggregates on the surface of preformed nanotoroids, increasing solvophobic interactions upon the alkyl chain extension enabled us to increase the catenation tendency. In the current system wherein, toroidal nanoaggregates are formed by the generation of intrinsic (spontaneous) curvature upon one-dimensional aggregation of the monomers, and the alkyl chain extension also resulted in a harsh steric restriction of secondary nucleation in nano-[2]catenane. Due to these two kinetic effects, nano-[2]catenane becomes the predominant topological species in a kinetic self-assembly process of 2 by an injection protocol. This unique method may be helpful to efficiently control the catenation tendency in the synthesis of polycatenanes using non-specific interaction. We also envisage that the catenation of sufficiently larger nanotoroids that could be realized by altering the alkyl chain length would provide branched nanocatenanes. Furthermore, creating heteromeric nano-[2]catenanes with donor and acceptor nanotoroids is an ongoing challenge in our group.

## Data availability

All supporting data is provided in the ESI.†

## Author contributions

S. Y. and H. I. designed the project. H. I. performed most of the experimental work except for SAXS fitting. S. Y. and H. I. prepared the overall manuscript, including figures. All authors, including S. D., R. T., and M. J. H., contributed by commenting on the manuscript. S. Y. supervised the overall research.

## Conflicts of interest

There are no conflicts to declare.

## Supplementary Material

SC-014-D2SC07063D-s001
