# Supplementary material for: Fine-tuning of the size of supramolecular nanotoroids suppresses the subsequent catenation of nano-[2]catenane
Source: Chem Sci. 2023 Mar 2;14(12):3270–6. doi: 10.1039/d2sc07063d (PMC10034040; doi:10.1039/d2sc07063d)
Supplement: SC-014-D2SC07063D-s001 [file SC-014-D2SC07063D-s001.pdf]

Electronic Supplementary Material (ESI) for Chemical Science.
This journal is © The Royal Society of Chemistry 2023

Electronic Supplementary Information

Fine-tuning of size of supramolecular nanotoroids suppresses subsequent catenation of nano-[2]catenane

Hiroki Itabashi,[a] Sougata Datta,[b] Ryohei Tsukuda,[a] Martin J. Hollamby,[c] and Shiki Yagai [b,d]

[a]Division of Advanced Science and Engineering, Graduate School of Science and Engineering, Chiba University, 1-33 Yayoi-cho, Inage-ku, Chiba 263-8522, Japan.
[b]Institute for Advanced Academic Research (IAAR), Chiba University, 1-33 Yayoi-cho, Inage-ku, Chiba 263-8522, Japan.
[c]Department of Chemistry, School of Chemical and Physical Sciences, Keele University, Keele, Staffordshire, ST55BG, UK.
[d] Department of Applied Chemistry and Biotechnology, Graduate School of Engineering, Chiba University, Chiba 263-8522, Japan.

**E-mail: yagai@faculty.chiba-u.jp*

This PDF file includes:

Supplementary Methods
Synthesis
Supplementary Figures
Supplementary References

Supplementary Methods

Materials

Compound **1** was synthesized by following previously reported procedure.[S1] Compound **2** was synthesized by following the procedures shown in scheme S1. All starting and reagents were purchased from commercial suppliers and utilized without further purification. Spectroscopic grade solvents were used for spectroscopic measurements without further purification.

Nuclear magnetic resonance (NMR) spectroscopy, atmospheric-pressure chemical ionization mass (APCI-MS) spectrometry

^{1}H and ^{13}C NMR spectra were recorded on Bruker AVANCE III-400M NMR spectrometer at 400MHz. ^{1}H NMR chemical shifts are reported in parts per million (ppm, δ) with the signal of tetramethylsilane (TMS) as internal standard at 0.00 ppm. The resonance multiplicity was represented in terms of s (singlet), d (doublet), t (triplet), brs (broad singlet) and m (multiplet). ^{13}C NMR chemical shifts reported in ppm (δ) were normalized with the chemical shifts of $CDCl_3$ at 77.16 ppm as reference. APCI-MS spectra measurements were conducted on an Exactive (Thermo Fisher).

UV-vis spectroscopy

UV-vis absorption spectra were measured by JASCO V660 and V760 spectrophotometers equipped with a JASCO ETCS-761 temperature-control units using a screw-capped quartz cuvette with 1.0 cm optical path-length.

Atomic force microscopy (AFM)

AFM imaging was carried out under ambient conditions using Multimode 8 Nanoscope V (Bruker Instrument) in Peak Force Tapping (ScanAsyst) mode. Silicon cantilevers (SCANASYST-AIR) with a spring constant of 0.4 N/m and frequency of 70 kHz (nominal value, Bruker, Japan) were used. The samples were prepared by spin-coating (3000 rpm, 1 min) of solutions (10 μL) of supramolecular polymers onto freshly cleaved highly oriented pyrolytic graphite (HOPG, 5 mm × 5 mm) at 293 K. Images were processed using NanoScope Analysis 1.40 (Bruker) and ImageJ v1.53s (National Institutes of Health).

Estimation of the relative area (%) of different topologies

For 500 × 500 nm^{2} AFM images of a particular sample, the total number of single and interlocked toroids was counted using the point tool of ImageJ. The total surface area occupied by a single toroid and all topologies (catenanes, single toroids and random coils, Figure S2) were measured using the Bearing Analysis mode of Nanoscope analysis software. The total surface area occupied by single toroids was calculated by multiplying the number of single toroids with the measured surface area of a single toroid, while the total surface area covered by catenanes was obtained by multiplying the number of interlocked toroids with the surface area of a single toroid. The relative surface area (%) of catenanes and single toroids were estimated with respect to the total surface area of all catenanes, single toroids

and random coils using the following equations.

Relative surface area of single toroid (%) =
100 × (Surface area of single toroids) / (Surface area occupied by all topologies)

Relative surface area of catenanes (%) =
100 × (Surface area of interlocked toroids) / (Surface area occupied by all topologies)

Relative surface area of catenanes (%) =
100 – (Relative surface area of single and interlocked toroids)

Small-Angle X-ray Scattering (SAXS)
SAXS experiments were carried out at BL-10C of the Photon Factory of the High Energy Accelerator Research Organization (KEK) in Tsukuba, Japan. Sample solutions were placed into 1.25-mm path length cells (20-μm thickness quartz glass windows surrounded by stainless steel), and the temperature was fixed at 293 K. X-ray with a wavelength of 1.5 Å and a sample-detector distance of 1029 mm (calibrated with silver behenate) resulted in a detectable Q range in the order of 0.1–5.9 nm^{-1}. Sixty frames were collected with each exposure time of 10 s. Because no radiation damage was observed, the collected data were averaged to give a total integration time of 600 s. The 2D scattering data (detector: DECTRIS PILATUS3 2M) were radially averaged to yield 1D scattering intensity data [$I(Q)$ vs. Q]. These data were then normalized using water as a reference, and the following subtraction of the background (quartz glass windows and solvent) gave absolute scattering intensity $I(Q)$ in cm^{-1}. All data reduction was performed using the software package SAngler.[S2]

Model fitting
Model fitting was carried out in much the same way as in previous related studies, using the SASFit[S3] data analysis software. The form factor for a torus with an elliptical cross-section, a scattering length density $\Delta\rho$, radius R, cross-sectional radius a and aspect ratio b is generally given as follows[S4]:

$$F_{torus}\ (Q, \Theta, R, a, b, \Delta\rho) = \int_{R-x}^{R+x} 4\pi\Delta\rho \frac{J_0(Qr\sin\Theta)\sin(Q\gamma(r)\cos\Theta)}{Q\cos\Theta} dr \qquad \text{(S1)}$$

$$\text{where } \gamma(r) = b\sqrt{a^2 - (r-R)^2} \qquad \text{(S2)}$$

In equation (S1), J_0 is the Bessel function of zero order. The overall scattering for a delta (monodisperse) distribution of core–shell toroids is then given as:

$$I_{torus+shell} = N \int_0^{\pi/2} |F_{torus}(Q, \Theta, R, a + \Delta a, b, \Delta\rho_{shell}) - F_{torus}(Q, \Theta, R, a, b, \Delta\rho_{core})|^2 \sin\Theta\, d\Theta \quad \text{(S3)}$$

i.e. for the shell from factor $F_{torus}(Q, \Theta, R, a + \Delta a, b, \Delta\rho_{shell})$, a in equations (S1) and (S2) is replaced with $(a + \Delta a)$, where Δa is the alkyl shell thickness, calculated using Tanford's formula[S5] for the length of an alkyl chain, adapted for a loss of a terminal C-H bond at one end ($l_c = 0.75 + 1.265\ n_c$, where n_c is the number of carbons). For both, $\Delta\rho$ in equation (S1) is replaced with $\Delta\rho_{core}$ or $\Delta\rho_{shell}$ for the core and shell form factor respectively, with $\rho_{core} = 12.6 \times 10^{-6}$ Å^{-2}, $\rho_{solvent} = 7.46 \times 10^{-6}$ Å^{-2} as previously shown[S6], and ρ_{shell} obtained by fitting. In equation S3, N is a scale factor that accounts for the number density of toroids. The assumption of a monodisperse delta distribution is a necessity: Incorporating polydispersity (PD) into the fitting isn't viable as the toroid model is complex and including PD, even in only R, would significantly increase the computational power required for the global fitting routine. Polydispersity tends to smear features, and even a moderate polydispersity would considerably smoothen the maxima and minima in the data. As the data shows well-defined features that are well-described by the monodisperse model, the toroids must have a low degree of polydispersity (in line with AFM observations).

The SAXS data arising from the toroid dispersions exhibit a noticeable increase in $I(Q)$ at low Q, which can be attributed to the formation of loose aggregates. Aggregation is likely to originate from the relatively high sample concentration that was required in order to obtain a sufficiently distinct scattering signal. In order to account for this signal at low Q, an attractive Ornstein-Zernike structure factor was used, which describes a decaying distribution, where ζ is a correlation length, and κ is related to the strength of interactions via the isothermal compressibility[S7]:

$$S(Q) = 1 + \left[\frac{\kappa}{1 + (Q\zeta)^2}\right] \quad \text{(S4)}$$

The total small-angle scattering signal was then calculated using equation S5. A very small flat background contribution, I_{bkg} was added to account for background scattering that wasn't able to be accounted for in the data reduction.

$$I(Q) = I_{torus+shell}S(Q) + I_{bkg} \quad \text{(S5)}$$

Preparation of toroid solution

A monomeric solution of **1** or **2** in chloroform ($c = 1.0 \times 10^{-3}$ M, 100 μL) was injected into 900 μL of MCH at r.t.. The resulting solution was heated at 363 K and 353 K for **1** and **2**, respectively, for 5 min and subsequently cooled to 293 K at a cooling rate of 1.0 K min^{-1}. The resulting solution was passed through a membrane filter of 200 nm pore size to filter out the elongated fibers from toroids (Fig. S3).

Estimation of degree of aggregation (*α*)

Using the temperature-dependent UV/vis absorption data, the degree of aggregation (α) at a given time (t) was calculated from Supplementary Equation:

$$\alpha(t) = \frac{\varepsilon(t) - \varepsilon_{\mathrm{mon}}}{\varepsilon_{\mathrm{agg}} - \varepsilon_{\mathrm{mon}}} \tag{S6}$$

where $\varepsilon_{\mathrm{agg}}$ and $\varepsilon_{\mathrm{mon}}$ are molar absorption coefficients at 470 nm of fully aggregated (the highest value, $\alpha = 1$) and pure monomeric species (the lowest value, $\alpha = 0$), respectively, and $\varepsilon(t)$ is the molar absorption coefficient at a given time.

Synthesis

Compound **2** was synthesized by following the procedure shown in Scheme S1. Compound **3**[S8] and **4**[S9] was synthesized according to previously reported methods.

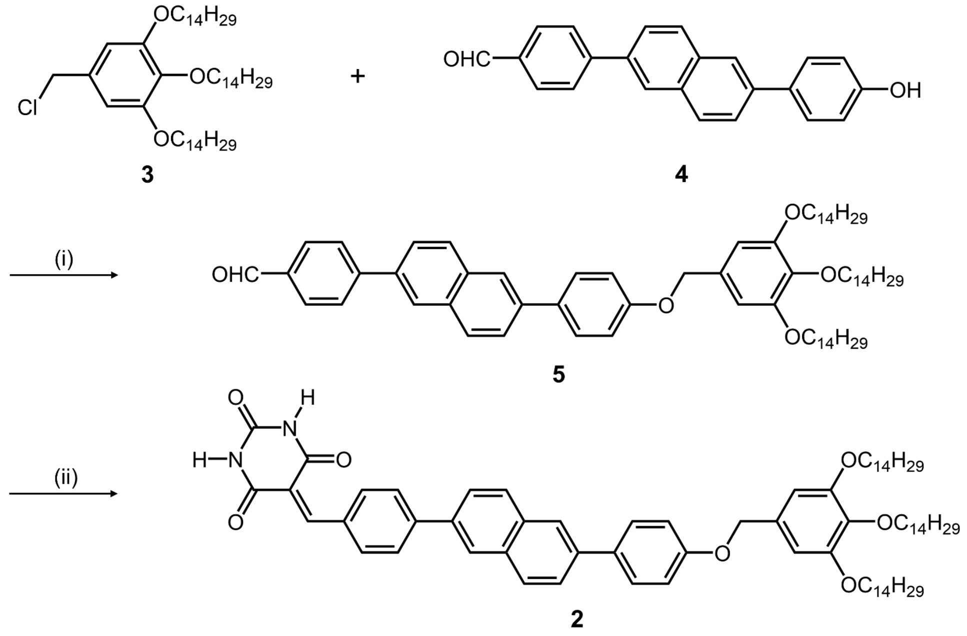

Scheme S1. Synthetic route of compound **2**. i) K_2CO_3, DMF, 343 K, 5 h; ii) barbituric acid, EtOH, reflux 24 h.

4-(6-(4-((3,4,5-Tris(tetradecyloxy)benzyl)oxy)phenyl)naphthalen-2-yl)benzaldehyde (5): A 50 mL three neck round bottom flask connected with refluxing condenser was charged with compound **4** (30 mg, 0.093 mmol), K_2CO_3 (24 mg, 0.17 mmol) and compound **3** (76 mg, 0.10 mmol). The flask was evacuated and refilled with N_2 gas three times. Dry DMF (10 mL) was added to the flask, and the mixture was stirred at 343 K for 5 h. After being cooled to r.t., the mixture was extracted with ethyl acetate:*n*-hexane (1:3, v/v) mixture, and the organic layer was washed with water. The organic layer was separated, dried over Na_2SO_4, and then evaporated to dryness under reduced pressure. The resulting solid was purified by column chromatography over silica gel (eluent: $CHCl_3$) to give compound **5** as white solids (85 mg, yield = 87%). ^{1}H NMR (400 MHz, 293 K, $CDCl_3$): δ 10.09 (s, 1H, C$\underline{H}$O), 8.12 (s, 1H), 8.03–7.96 (m, 5H), 7.91 (d, 2H, J = 8.3 Hz), 7.78 (d, 2H, J = 8.4 Hz), 7.69 (d, 2H, J = 8.4 Hz), 7.11 (d, 2H, J = 8.4 Hz), 6.66 (s, 2H), 5.02 (s, 2H), 4.01–3.94 (m, 6H, OC$\underline{H}_2$), 1.84–1.73 (m, 6H), 1.48–1.26 (m, 66H), 0.89–0.86 (m, 9H) ppm. ^{13}C NMR (100 MHz, 293 K, $CDCl_3$): δ 191.96, 158.67, 153.37, 147.10, 138.90, 138.01, 136.74, 135.19, 133.54, 133.49, 132.42, 131.76, 130.39, 128.97, 128.91, 128.43, 127.86, 126.37, 126.19, 125.54, 124.77, 115.36, 106.16, 73.47, 70.59, 69.16, 31.96, 30.37, 29.78, 29.74, 29.70, 29.66, 29.59, 29.46,

29.43, 29.40, 26.17, 26.14, 22.72, 14.16 ppm. HRMS (APCI): *m*/*z* calcd for $C_{72}H_{107}O_5$ 1051.8124 $[M+H]^+$, found 1051.8113.

5-(4-(6-(4-((3,4,5-Tris(tetradecyloxy)benzyl)oxy)phenyl)naphthalen-2-yl)benzylidene)pyrimidine-2,4,6(1H,3H,5H)-trione (**2**): A mixture of **5** (31 mg, 0.030 mmol) and barbituric acid (21 mg, 0.16 mmol) in EtOH (7 mL) was refluxed for 24 h. The reaction mixture was cooled to r.t., and the resulting precipitates were collected by filtration and washed with hot EtOH repeatedly. The residual solid was further purified by reprecipitation from a $CHCl_3$-MeOH mixture to give pure compound **2** as orange solids (29 mg, yield = 83%). ^1H NMR (400 MHz, 293 K, $CDCl_3$): δ = 8.65 (s, 1H), 8.38 (d, 2H, J = 8.6 Hz), 8.17 (brs, 1H), 8.03 (brs, 1H), 8.00–7.97 (m, 3H), 7.90 (d, 3H, J = 8.5 Hz), 7.83–7.77 (m, 2H), 7.69 (d, 2H, J = 8.6 Hz), 7.11 (d, 2H, J = 8.7 Hz), 6.66 (s, 2H), 5.03 (s, 2H), 4.01–3.94 (m, 6H, $OC\underline{H}_2$), 1.82–1.73 (m, 6H), 1.47–1.25 (m, 66H), 0.89–0.86 (m, 9H) ppm. ^{13}C NMR (100 MHz, 333 K, $CDCl_3$): δ 162.66, 160.41, 159.7, 158.87, 153.45, 148.31, 146.69, 139.13, 138.7, 136.48, 135.66, 133.73, 133.63, 132.51, 131.85, 131.09, 128.94, 128.35, 127.09, 126.34, 126.15, 125.23, 124.73, 115.58, 106.75, 73.45, 70.67, 69.51, 31.85, 30.36, 30.19, 29.93, 29.65, 29.62, 29.57, 29.51, 29.38, 29.25, 26.12, 26.09, 22.56, 13.89 ppm. HRMS (APCI): *m*/*z* calcd for $C_{76}H_{109}O_7N_2$ = 1161.8223 $[M+H]^+$, found 1161.8229.

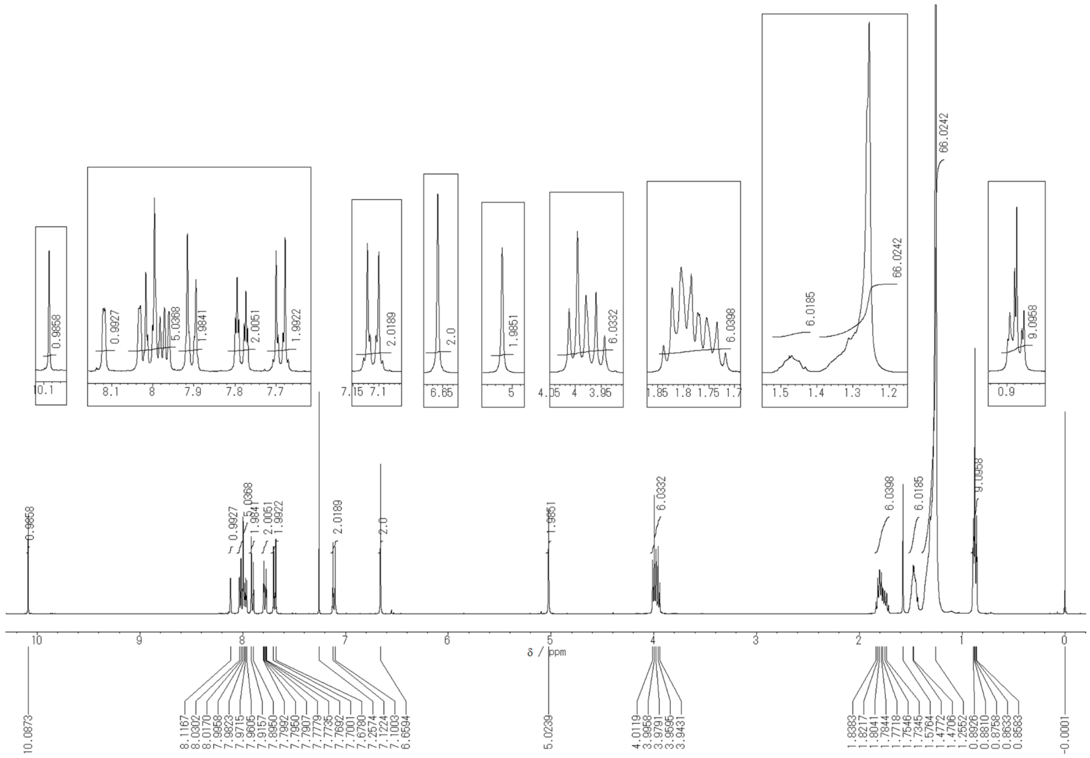

Chart S1 ^{1}H NMR spectrum of compound **5** in $CDCl_3$ at 293 K.

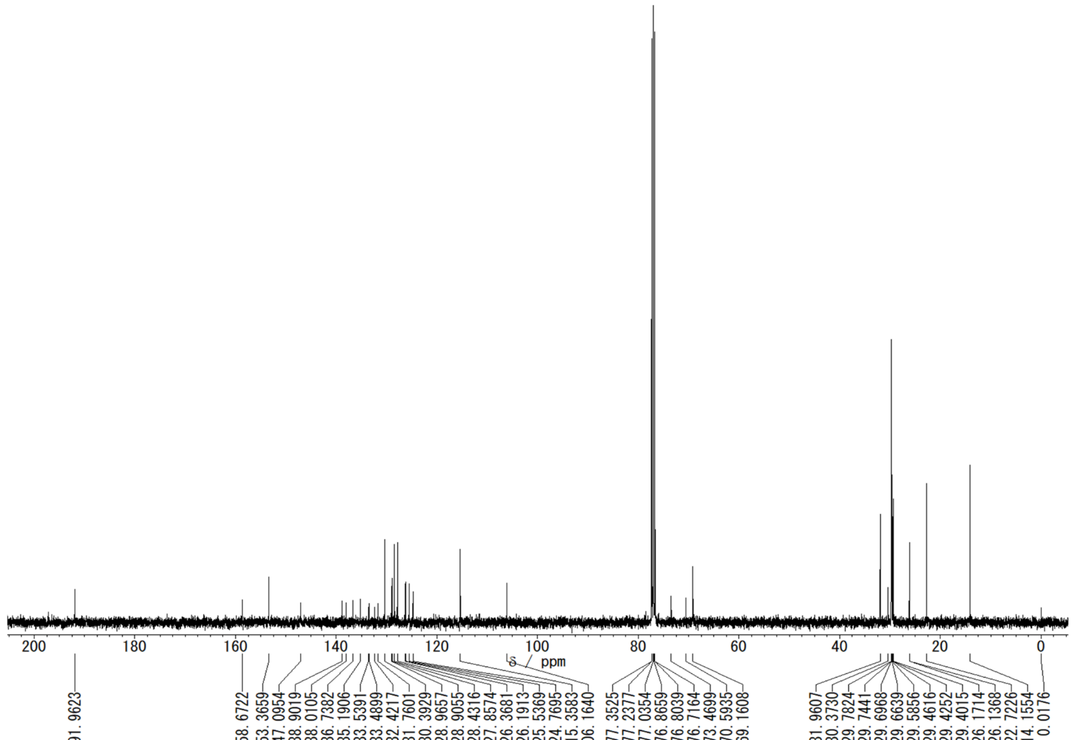

Chart S2 ^{13}C NMR spectrum of compound **5** in $CDCl_3$ at 293 K.

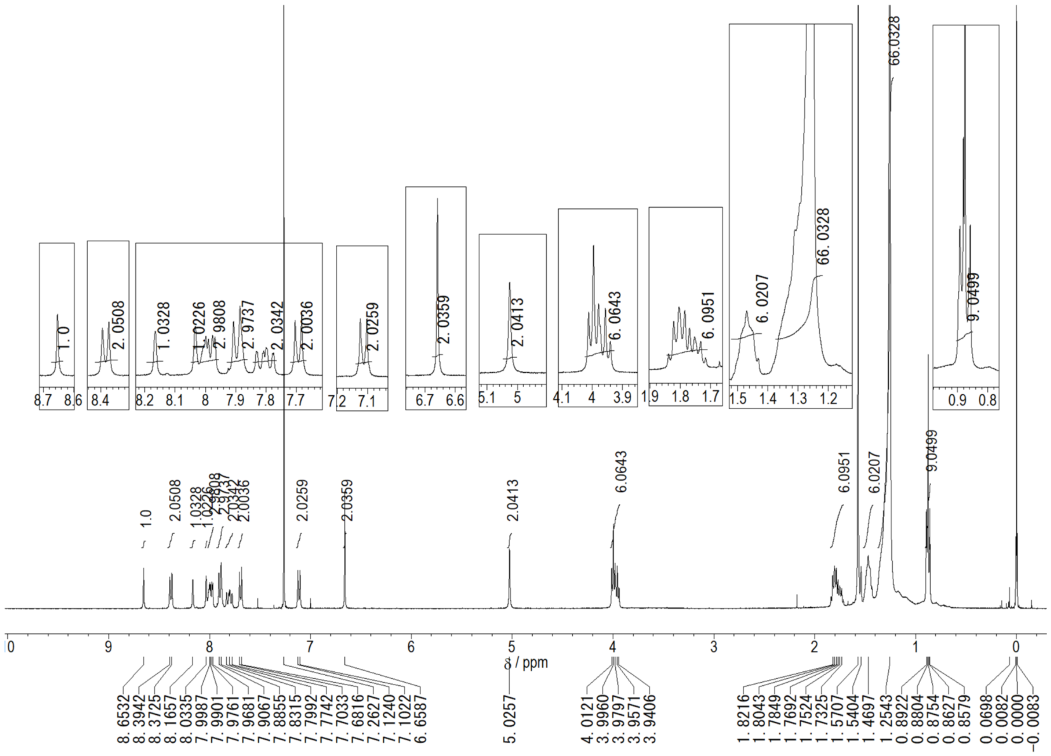

Chart S3 ^{1}H NMR spectrum of compound **2** in $CDCl_3$ at 293 K.

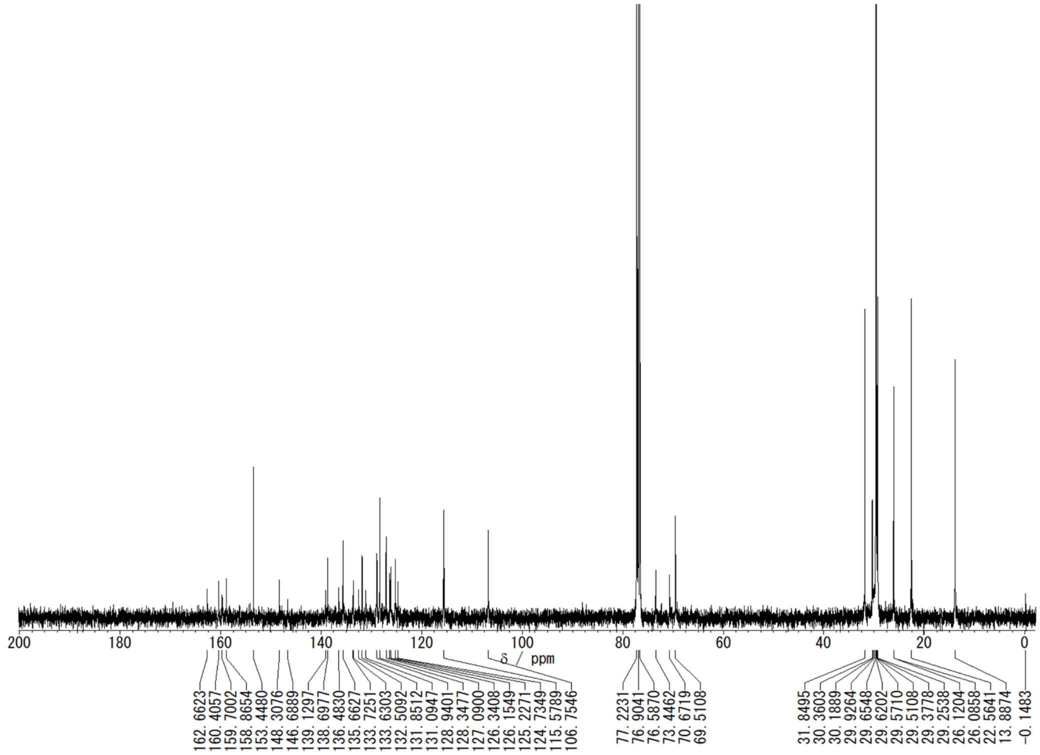

Chart S4 ^{13}C NMR spectrum of compound **2** in $CDCl_3$ at 333 K.

Supplementary Figures

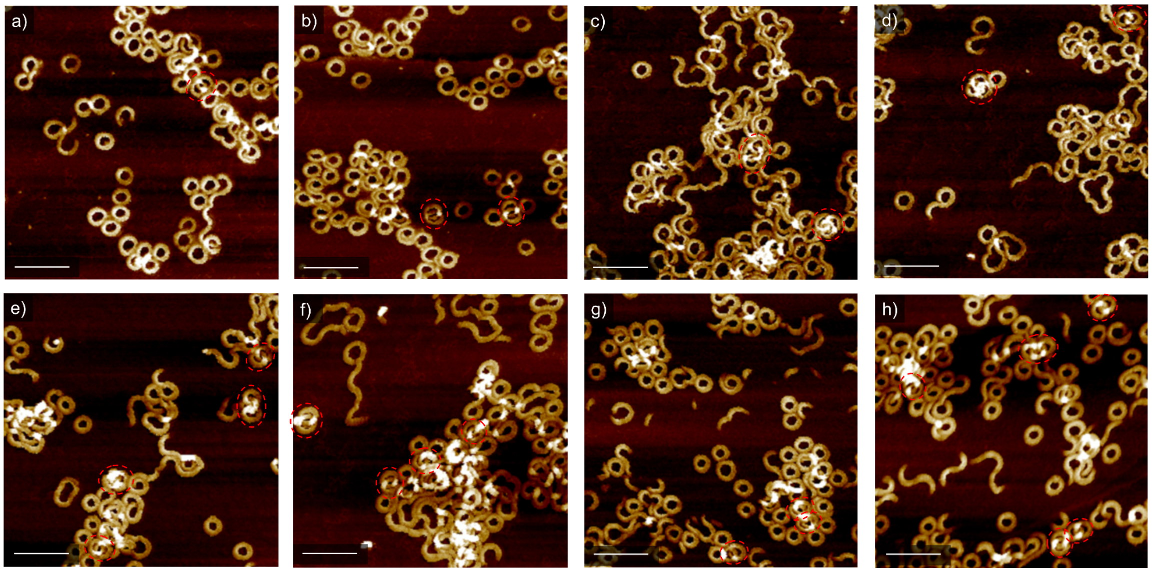

Fig. S1 AFM images of supramolecular polymers obtained by injecting a 100 μL chloroform solution (c = 1.0 × 10^{-3} M) of **1** (a–d) or **2** (e–h) into 900 μL of MCH in one portion. Scale bars, 100 nm. Catenated toroids are shown by dotted red circles.

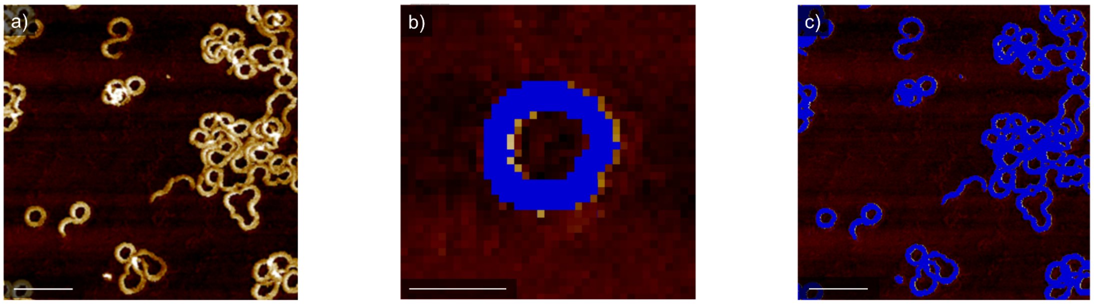

Fig. S2 (a) AFM image of supramolecular polymers prepared by injecting a 100 μL chloroform solution (c = 1.0 × 10^{-3} M) of **1** into 900 μL of MCH in one portion. Scale bar, 100 nm. (b) The surface area of a single toroid in (a). Scale bar, 25 nm. (c) The total surface area occupied by all topologies in (a). Scale bar, 100 nm. These surface areas were measured by the Bearing Analysis mode of a Nanoscope analysis software.

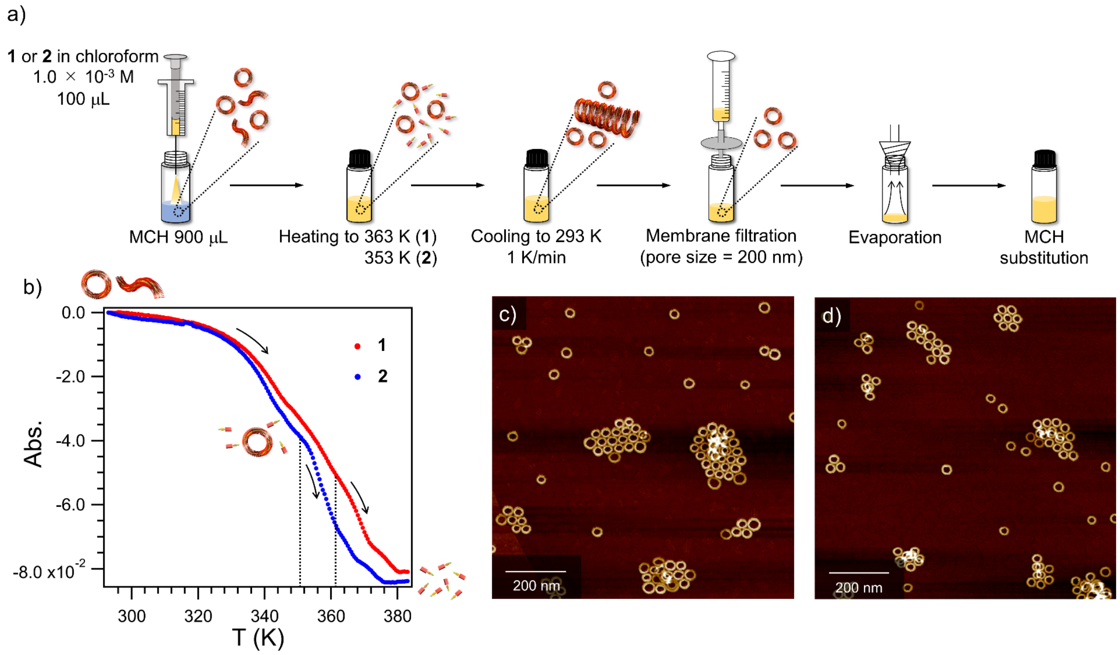

Fig. S3 (a) Depiction of the protocol to separation of toroids from a mixture of open-ended fibers. (b) Heating curves at λ = 470 nm of supramolecular polymers obtained by injecting 100 μL chloroform solution ($c = 1.0 \times 10^{-3}$ M) of **1** (red) or **2** (blue) into 900 μL of MCH in one portion. Toroids of **1** and **2** begin to disassemble around 363 and 353 K (shown by dotted lines), respectively. (c, d) AFM images of the isolated toroids of **1** (c) and **2** (d).

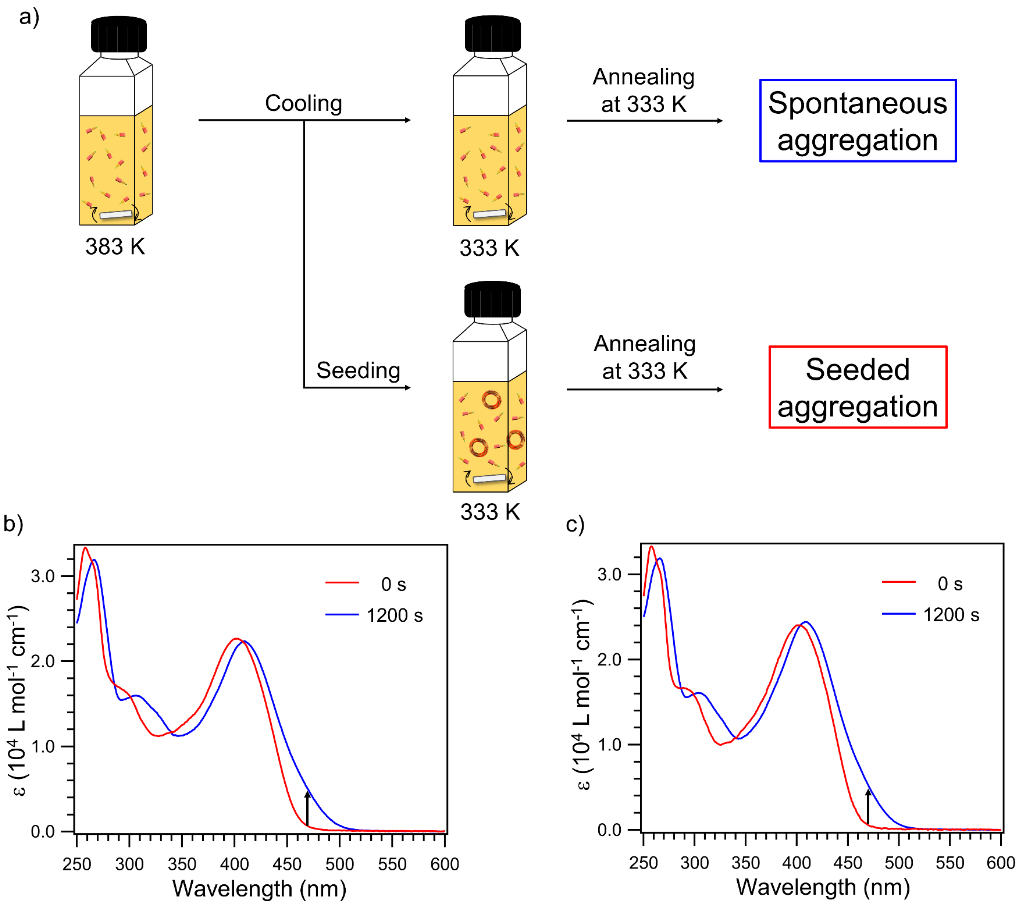

Fig. S4 (a) Schematic illustration of spontaneous and seeded supramolecular polymerization processes of **1** and **2**. (b, c) Time-dependent changes in the UV-Vis spectra of unseeded MCH solutions of **1** (b) (c = 7.5 × 10^{-6} M) and **2** (c) (c = 9.0 × 10^{-6} M) at 333 K. *Note:* In a typical seeding experiment, 100 μl of the MCH solution containing toroid seeds was added to a monomer solution (3 ml) in MCH at 353 K. The final concentrations of the monomer were 7.5 × 10^{-6} M and 7.6 × 10^{-6} M for **1** and **2**, respectively and those of the seed were 2.4 × 10^{-6} M for both **1** and **2**. Then, the kinetics of the seeded supramolecular polymerization of **1** and **2** were studied at 333 K by UV–Vis spectroscopy to follow the growth of a new absorption band at λ = 470 nm, which is attributed to π–π stacking of the diphenylnaphthalene core.[S1] To obtain reproducible kinetic data, in the present study we applied stirring during annealing the solutions at 333 K.[S6]

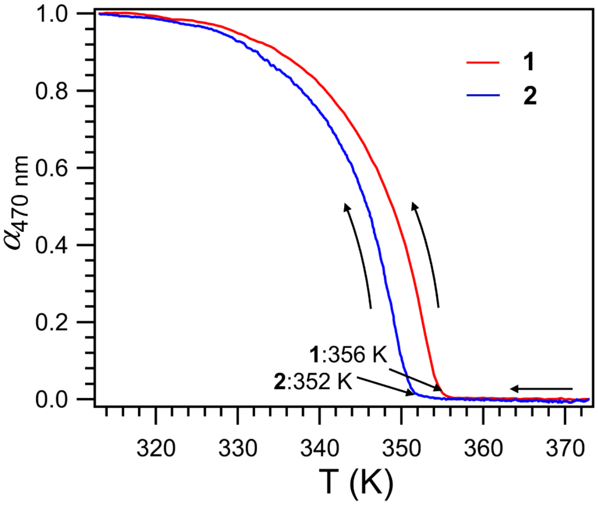

Fig. S5 Cooling curves of **1** (red) and **2** (blue) obtained by temperature-dependent absorption measurements at 470 nm in MCH at 20 μM. The cooling rate was 1 K/min.

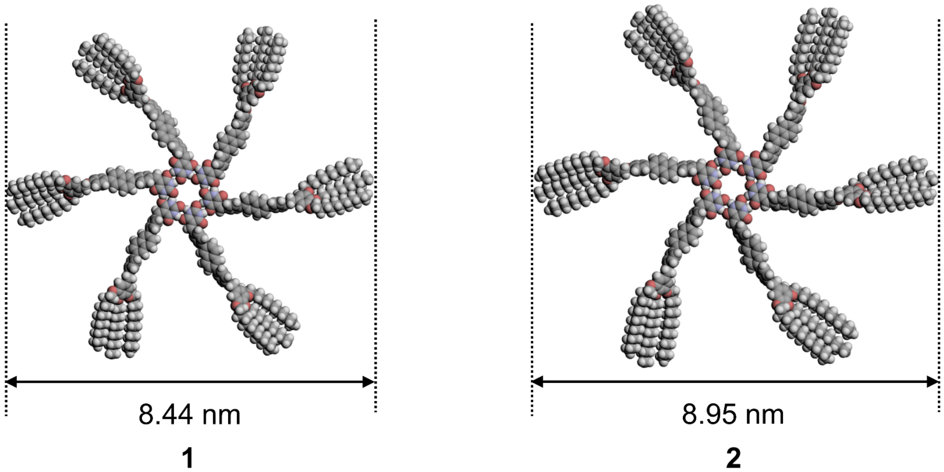

Fig. S6 Geometry-optimized structures of rosettes of **1** and **2**. The geometry optimization by the molecular mechanics calculation was performed on MacroModel/Maestro version 12.2 (Schrödinger) with AMBER* force field in the absence of solvent.

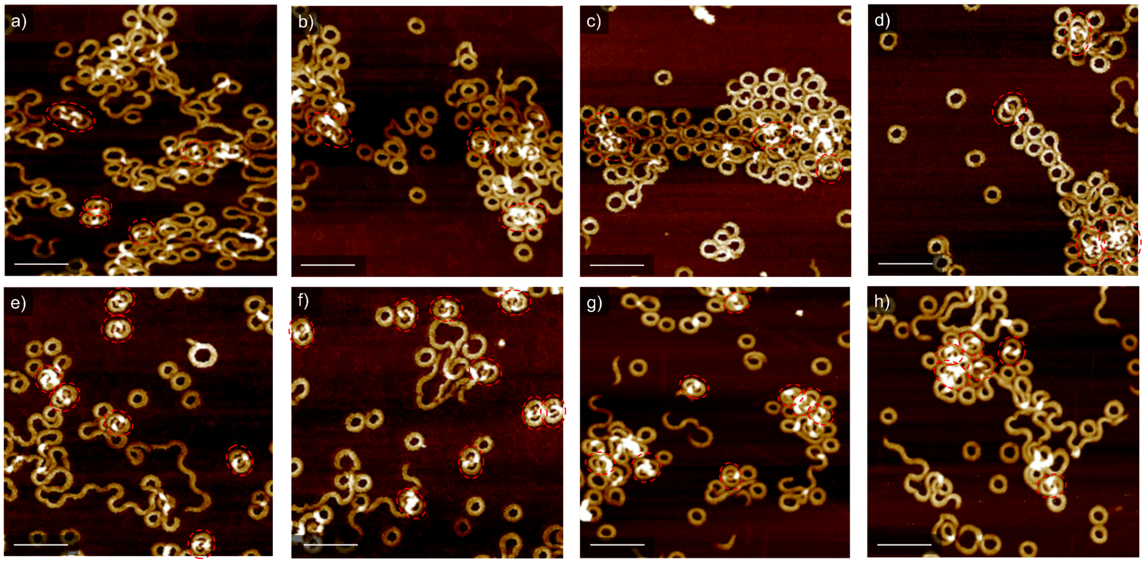

Fig. S7 AFM images of supramolecular polymers obtained by injecting a 100 μL chloroform solution (c = 1.0 × 10^{-3} M) of **1** (a–d) or **2** (e–h) into 900 μL of MCH in ten portions (one injection per second). Scale bars, 100 nm. Catenated toroids are surrounded by dotted red circles.

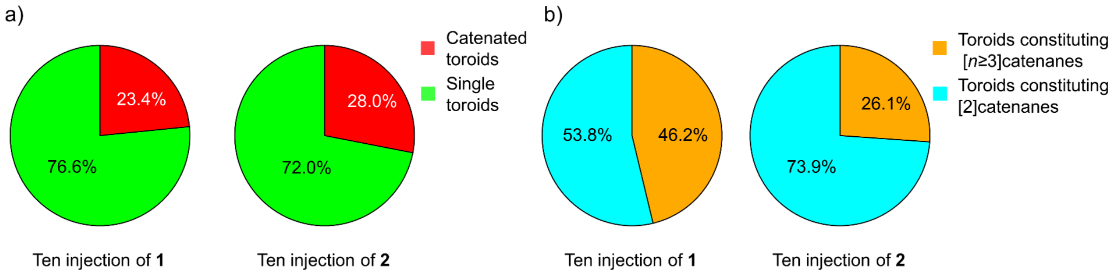

Fig. S8 (a) Pie charts showing the percentage of interlocked toroids constituting various nano-[n]catenanes (red) and single toroids (green) obtained by injecting a 100 μL chloroform solution (c = 1.0 × 10^{-3} M) of **1** or **2** into 900 μL of MCH in ten portions (one injection per second). (b) Pie charts showing the percentage of interlocked toroids constituting nano-[2]catenane (blue) and oligomeric nano-[n≥3]catenanes (orange) obtained by injecting a 100 μL chloroform solution (c = 1.0 × 10^{-3} M) of **1** or **2** into 900 μL of MCH in ten portions.

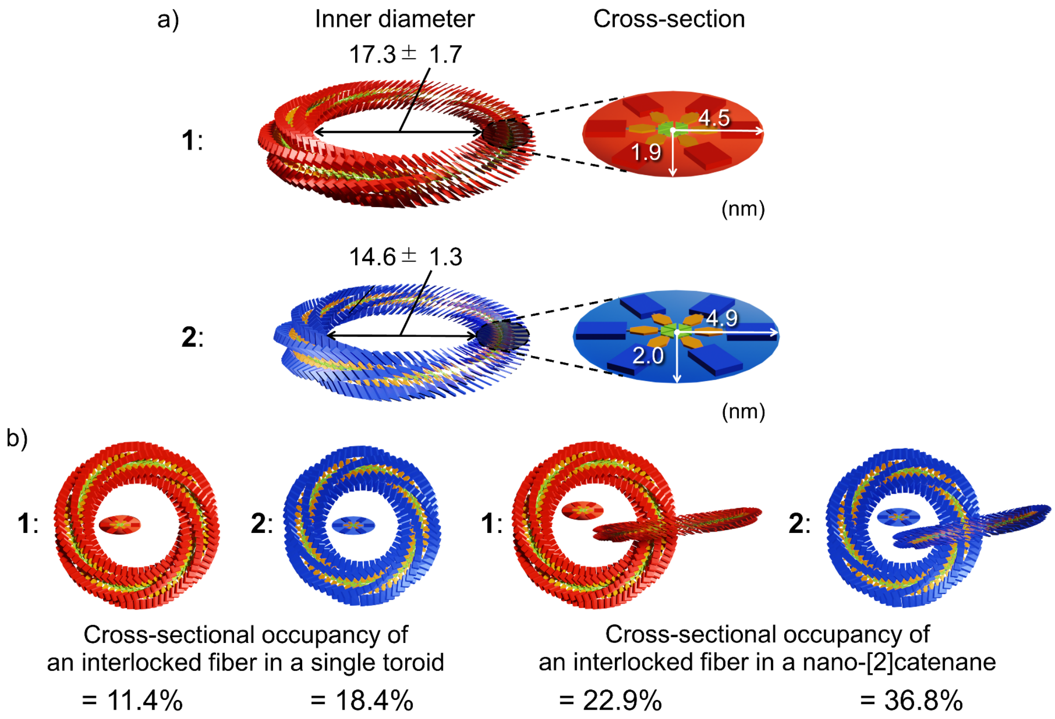

Fig. S9 (a) Cartoon representations of sizes of toroids and rosettes of **1** (red) and **2** (blue). (b) Representations of occupancy of an interlocked fiber of **1** (red) and **2** (blue) in the corresponding single toroid and nano-[2]catenane based on AFM cross-sectional data, as shown in Fig. 3a and 3b.

Table S1 Dimensions of toroids of **1** and **2** measured by AFM and SAXS.

Measured by AFM	**1** (nm)	**2** (nm)
Diameter	26.3 ± 1.7	24.3 ± 1.4
Inner diameter	17.3 ± 1.7	14.6 ± 1.3
Fiber width	9.0 ± 1.7	9.8 ± 1.3
Fiber height	3.8 ± 0.4	4.0 ± 0.4

Measured by SAXS	**1** (nm)	**2** (nm)
Diameter	26.2 ± 0.4	25.6 ± 0.2
Inner diameter	18.0 ± 0.4	16.8 ± 0.2
Fiber width	8.2	8.8
Fiber height	4.8	5.3

Supporting Reference

S1 D. D. Prabhu, K. Aratsu, Y. Kitamoto, H. Ouchi, T. Ohba, M. J. Hollamby, N. Shimizu, H. Takagi, R. Haruki, S. Adachi and S. Yagai, *Sci. Adv.*, 2018, **4**, eaat8466.

S2 N. Shimizu, K. Yatabe, Y. Nagatani, S. Saijyo, T. Kosuge and N. Igarashi, *AIP Conf. Proc.*, 2016, **1741**, 050017.

S3 I. Breβler, J. Kohlbrecher and A. F. Thünemann, *J. Appl. Cryst.*, 2015, **48**, 1587–1598.

S4 T. Kawaguchi, *J. Appl. Cryst.*, 2001, **34**, 580–584.

S5 C. Tanford *The hydrophobic effect: formation of micelles and biological membranes*, Wiley, New York, 1980.

S6 S. Datta, Y. Kato, S. Higashiharaguchi, K. Aratsu, A. Isobe, T. Saito, D. D. Prabhu, Y. Kitamoto, M. J. Hollamby, A. J. Smith, R. Dalgliesh, N. Mahmoudi, L. Pesce, C. Perego, G. M. Pavan and S. Yagai, *Nature*, 2020, **583**, 400–405.

S7 M. Kotlarchyk, S.-H. Chen, J. S. Huang and M. W. Kim, *Phys, rev, A*, 1984, **29**, 2054–2069.

S8 V. Percec, M. Peterca, Y. Tsuda, B. M. Rosen, S. Uchida, M. R. Imam, G. Unger and P. A. Heiney, *Chem. Eur. J.*, 2009, **15**, 8994–9004.

S9 T. Aizawa, S. Takahashi, A. Isobe, S. Datta, H. Sotome, H. Miyasaka, T. Kajitani and S. Yagai, *Chem. Lett.*, 2020, **49**, 1009–1012.
